# International pilot external quality assessment scheme for analysis and reporting of circulating tumour DNA

**DOI:** 10.1186/s12885-018-4694-x

**Published:** 2018-08-09

**Authors:** Cleo Keppens, Elisabeth M. C. Dequeker, Simon J. Patton, Nicola Normanno, Francesca Fenizia, Rachel Butler, Melanie Cheetham, Jennifer A. Fairley, Hannah Williams, Jacqueline A. Hall, Ed Schuuring, Zandra C. Deans, On behalf of IQN Path ASBL

**Affiliations:** 1 Department of Public Health and Primary Care, Biomedical Quality Assurance Research Unit, University of Leuven, Kapucijnenvoer 35d, 3000 Leuven, Belgium; 2 European Society of Pathology (ESP), Anderlecht, Belgium; 3 European Molecular Quality Network (EMQN), Manchester Centre for Genomic Medicine, St Mary’s Hospital, Manchester, M13 9WL UK; 4 Cell Biology and Biotherapy Unit, Instituto Nazionale Tumori “Fondazione Giovanni Pascale”, IRCCS, Napoli, Italy; 5 All Wales Genetic Laboratory, Institute of Medical Genetics, University Hospital of Wales, Heath Park, Cardiff, CF14 4XW UK; 6 UK NEQAS for Molecular Genetics, Department of Laboratory Medicine, Royal Infirmary of Edinburgh, Little France Crescent, Edinburgh, EH16 4SA UK; 7 International Quality Network for Pathology (IQN Path) Association Sans But Lucratif (A.S.B.L), 3A Sentier de l’Espérance, L-1474 Luxembourg City, Luxembourg; 8 Division of Cancer, Department of Surgery and Cancer, Imperial College London, London, UK; 9 Department of Pathology, University of Groningen, University Medical Center of Groningen, Groningen, the Netherlands

**Keywords:** *KRAS*, *NRAS*, *EGFR*, Mutation testing, ctDNA, cfDNA, Lung cancer, Colorectal cancer

## Abstract

**Background:**

Molecular analysis of circulating tumour DNA (ctDNA) is becoming increasingly important in clinical treatment decisions. A pilot External Quality Assessment (EQA) scheme for ctDNA analysis was organized by four European EQA providers under the umbrella organization IQN Path, in order to investigate the feasibility of delivering an EQA to assess the detection of clinically relevant variants in plasma circulating cell-free DNA (cfDNA) and to analyze reporting formats.

**Methods:**

Thirty-two experienced laboratories received 5 samples for *EGFR* mutation analysis and/or 5 samples for *KRAS* and *NRAS* mutation analysis. Samples were artificially manufactured to contain 3 mL of human plasma with 20 ng/mL of fragmented ctDNA and variants at allelic frequencies of 1 and 5%.

**Results:**

The scheme error rate was 20.1%. Higher error rates were observed for *RAS* testing when compared to *EGFR* analysis, for allelic frequencies of 1% compared to 5%, and for cases including 2 different variants. The reports over-interpreted wild-type results and frequently failed to comment on the amount of cfDNA extracted.

**Conclusions:**

The pilot scheme demonstrated the feasibility of delivering a ctDNA EQA scheme and the need for such a scheme due to high error rates in detecting low frequency clinically relevant variants. Recommendations to improve reporting of cfDNA are provided.

**Electronic supplementary material:**

The online version of this article (10.1186/s12885-018-4694-x) contains supplementary material, which is available to authorized users.

## Background

In the last decade, the analysis of predictive biomarkers has become an essential step in the optimisation of therapy for cancer patients [1, 2] In routine practice, tumour-specific mutation testing entails the analysis of DNA extracted from tumour tissue which is harvested from resections or biopsies. However, tumour tissue sampling is often difficult, especially in patients with advanced disease. In some cases, the tumour sample can yield insufficient DNA for molecular analysis. This is particularly evident in non-small-cell lung cancer (NSCLC) patients, where in approximately 30% of patients a tissue sample is not available for epidermal growth factor receptor (*EGFR*) mutation analysis, either at diagnosis or as the disease progresses [3]. In these cases, the analysis of circulating cell-free (cfDNA) derived from plasma has been proposed as an alternative method for mutation testing [4, 5].

Plasma-derived cfDNA contains both circulating tumour DNA (ctDNA) and nucleic acids released by normal dividing cells. The mechanism by which tumour cells release ctDNA into the blood is not fully known. It is thought to involve mechanisms such as apoptosis and necrosis, as suggested by the specific fragmentation pattern of ctDNA (+/− 160 base pairs) which in turn is suggestive of a nuclease-dependent degradation [6, 7]. It has also been proposed that tumour cells may secrete DNA fragments through vesicles [3].

The advantage of cfDNA testing is that it is minimally invasive and avoids incomplete or variable results arising from tumour heterogeneity [8]. It may also be used to monitor tumour progression [4, 5]. Many studies have demonstrated the effectiveness of assessing tumour-specific alterations by testing plasma cfDNA. This evidence led the European Medicine Agency to approve the use of plasma to detect *EGFR* mutations in the plasma of patients with advanced NSCLC, when adequate tissue is not available [9–11].

In patients with metastasized colorectal cancer (CRC), cfDNA testing for Kirsten rat sarcoma viral oncogene homolog (*KRAS*) and neuroblastoma rat sarcoma viral oncogene homolog (*NRAS*) mutations also holds prognostic value [12]. Consequently, numerous diagnostic tools for the detection of *EGFR*, *KRAS*, *NRAS* and *BRAF* mutations in cfDNA have recently become available. Subsequently the role of cfDNA has moved from use in diagnostic research to becoming a relevant testing matrix in patients with solid tumours [13]. However, the introduction of this novel methodology into clinical practice can be challenging for many laboratories. For instance, the standardization of testing procedures is complex, ranging from plasma collection, cfDNA extraction and cfDNA mutation analysis, to result interpretation. In addition, the analysis must be sufficiently sensitive to identify rare mutant molecules in a background of wild-type DNA at range of 0.1–1%. Currently, clinical applications of cfDNA are focused on the identification of primary mutations in pretreatment samples and the subsequent detection of resistant mutations upon progression in longitudinal samples, which inform treatment decisions. However, the potential uses are numerous and could include tumour monitoring and early tumour diagnosis [4].

The objectives of this External Quality Assessment (EQA) pilot scheme were to (i) investigate the feasibility of designing and delivering a technically challenging EQA (ii) evaluate and compare the ability of laboratories to detect cfDNA in plasma samples (iii) evaluate which extraction methodologies and testing method strategies were used and (iv) to assess the reporting of ctDNA testing results. For this purpose, four European EQA providers (Associazione Italiana di Oncologia Medica – AIOM, European Molecular Genetics Quality Network - EMQN, European Society of Pathology – ESP, United Kingdom National External Quality Assessment Service (UK NEQAS) for Molecular Genetics under the umbrella organization the International Quality Network for Pathology (IQN Path) [14], organised a pilot ctDNA EQA scheme. In this paper, we present the results of this scheme for the analysis of cfDNA for clinically relevant mutations as well as provide recommendations for reporting.

## Methods

### EQA scheme design

The pilot EQA was developed in 2016 and delivered to participants during 2017 as a collaboration between the four EQA providers. It was co-ordinated under the banner of an IQN Path working group, with additional expertise provided by scientific advisors. The pilot was carried out according to the requirements of the International Standard for Conformity assessment of proficiency testing ISO 17043 [15] to ensure a robust audit trail was associated with its design, development and implementation.

Thirty-two participant laboratories (eight from each EQA provider) were chosen from a pool of 167 potential candidates who completed a selection survey [13]. Selection criteria included technology available (to ensure material suitability for a range of different technologies), clinical diagnostic workload (to ensure inclusion of laboratories delivering a clinical ctDNA testing service), global location (to assess sample stability during transportation) and testing for *EGFR* and/or *RAS* genes (to ensure relevance to current clinical practice).

The pilot EQA scheme consisted of a set of eight samples containing mutations in the *EGFR, KRAS* or *NRAS* genes, in addition to two wild-type samples. The samples were shipped on dry ice (BioCair, Cambridge, United Kingdom) to each participant laboratory and the transit temperatures were monitored. Participants were asked to test the samples for the isolation of cfDNA and subsequent genotyping according to their established routine procedures. A central system for electronic result collection was set up in accordance with ISO 17043 [15] to which the validating laboratories as well as the participants were able to submit their genotyping results and background information on the testing process.

Participating laboratories were asked to submit interpretative diagnostic reports for assessment via their EQA provider. All results provided within the submitted reports were assessed independently by at least two IQN Path working group members against the same pre-defined scoring criteria, harmonized between the four EQA providers (Table 1). Samples A-E versus samples F-J were scored, for *RAS* and *EGFR* testing, respectively. For every case, a maximum of 2 points was awarded and points were deducted depending on the type of error made (Table 1). This yielded a total genotyping score on 20 points for participants to both *RAS* and *EGFR* analysis, and a total score on 10 points for participants to one of both sample sets. For every case, an average genotyping score was calculated on the maximum of 2 points across all participants. Each participant laboratory received an individual feedback report (Additional file 1), as well as a general report summarizing the expected results, scheme statistics and final results.

**Table 1:** Average genotyping score, assigned score criteria and error rates for every case analysed by the pilot scheme participants

Sample	A	B	C	D	E	F	G	H	I	J	A-J
Variant status	5% *KRAS* c.35G>C; p.(G12A)	1% *KRAS* c.35G>C; p.(G12A)	5% *NRAS* c.182A>G; p.(Q61R)	1% *NRAS* c.182A>G; p.(Q61R)	*KRAS/NRAS* Wild-type	5% *EGFR* c.2235_2249del15; p.E746_A750del	1% *EGFR* c.2235_2249del15; p.E746_A750del	5% *EGFR* c.2573T>G; p.(L858R) and c.2369C>T; p.(T790M)	1% *EGFR* c.2573T>G; p.(L858R) and c.2369C>T; p.(T790M)	*EGFR* Wild-type	Overall
Average score per sample on 2 points	1.5	1.3	1.7	1.5	2.0	1.7	1.7	1.9	1.6	1.9	1.7
Score criteria	# obtained scores (%)										
Correct (2 points)	11 (35.5%)	7 (22.6%)	12 (38.7%)	8 (25.8%)	23 (74.2%)	5 (16.1%)	4 (12.9%)	29 (93.5%)	18 (58.1%)	30 (96.8%)	147 (47.4%)
Correct but unspecified (2 points)	5 (16.1%)	3 (9.7%)	4 (12.9%)	2 (6.5%)	0 (0.0%)	16 (51.6%)	16 (51.6%)	0 (0.0%)	0 (0.0%)	0 (0.0%)	46 (14.8%)
False-negative ≤ LOD (2 points)^a^	0 (0.0%)	5 (16.1%)	0 (0.0%)	4 (12.9%)	0 (0.0%)	0 (0.0%)	2 (6.5%)	0 (0.0%)	6 (19.4%)	0 (0.0%)	17 (5.5%)
False-negative but variant not tested for (2 points)^a^	0 (0.0%)	0 (0.0%)	3 (9.7%)	3 (9.7%)	0 (0.0%)	0 (0.0%)	0 (0.0%)	0 (0.0%)	0 (0.0%)	0 (0.0%)	6 (1.9%)
False-negative >/unknown LOD (0 points)^a^	1 (3.2%)	5 (16.1%)	3 (9.7%)	4 (12.9%)	0 (0.0%)	0 (0.0%)	1 (3.2%)	0 (0.0%)	3 (9.7%)	0 (0.0%)	17 (5.5%)
False-positive (0 points)^a^	2 (6.5%)	2 (6.5%)	0 (0.0%)	0 (0.0%)	0 (0.0%)	0 (0.0%)	1 (3.2%)	0 (0.0%)	0 (0.0%)	1 (3.2%)	6 (1.9%)
Incorrect mutation detected with therapeutic implications (0 points)^a^	0 (0.0%)	0 (0.0%)	1 (3.2%)	1 (3.2%)	0 (0.0%)	0 (0.0%)	0 (0.0%)	0 (0.0%)	0 (0.0%)	0 (0.0%)	2 (0.6%)
Incorrect mutation detected without therapeutic implications (2 points)	3 (9.7%)	1 (3.2%)	0 (0.0%)	1 (3.2%)	0 (0.0%)	0 (0.0%)	0 (0.0%)	0 (0.0%)	0 (0.0%)	0 (0.0%)	5 (1.6%)
One variant missed in double mutation sample (0 points)^a^	N/A	N/A	N/A	N/A	N/A	N/A	N/A	2 (6.5%)	4 (12.9%)	N/A	6 (1.9%)
Mutation described incorrectly (1 point)	0 (0.0%)	0 (0.0%)	0 (0.0%)	0 (0.0%)	0 (0.0%)	10 (32.3%)	6 (19.4%)	0 (0.0%)	0 (0.0%)	0 (0.0%)	16 (5.2%)
Technical failure (not scored)	1 (3.2%)	0 (0.0%)	0 (0.0%)°	0 (0.0%)°	0 (0.0%)	0 (0.0%)	1 (3.2%)	0 (0.0%)	0 (0.0%)	0 (0.0%)	2 (0.6%)
Not tested (not scored)	8 (25.8%)	8 (25.8%)	8 (25.8%)	8 (25.8%)	8 (25.8%)	0 (0.0%)	0 (0.0%)	0 (0.0%)	0 (0.0%)	0 (0.0%)	40 (12.9%)
Total scored (*n* = 31)	22 (71.0%)	23 (74.2%)	23 (74.2%)	23 (74.2%)	23 (74.2%)	31 (100.0%)	30 (96.8%)	31 (100.0%)	31 (100.0%)	31 (100.0%)	268 (86.5%)
Error rate	# obtained scores (%)										
Total with implication on therapy decision^a^	3 (13.6%)	12 (52.2%)	7 (30.4%)	12 (52.2%)	0 (0.0%)	0 (0.0%)	4 (13.3%)	2 (6.5%)	13 (41.9%)	1 (3.2%)	54 (20.1%)
*Gene not tested/No method info given*	*0*	*0*	*3*	*3*	*0*	*0*	*0*	*0*	*0*	*0*	*6*
*NGS*	*2*	*7*	*3*	*7*	*0*	*0*	*1*	*1*	*7*	*1*	*29*
*Commercial Kit*	*0*	*3*	*1*	*1*	*0*	*0*	*0*	*0*	*1*	*0*	*6*
*LDT*	*1*	*1*	*0*	*0*	*0*	*0*	*1*	*0*	*1*	*0*	*4*
*BEAMing*	*0*	*0*	*0*	*0*	*0*	*0*	*0*	*0*	*0*	*0*	*0*
*ddPCR*	*0*	*1*	*0*	*1*	*0*	*0*	*2*	*1*	*4*	*0*	*9*

*N/A* Not applicable

^a^Values were used to calculate the error rates. °One partial technical failure for *NRAS,* only correct *KRAS* WT status was assessed on these cases. Reference sequence at time of scoring: *EGFR*: NM_005228.4 or LRG_304t1; *KRAS*: NM_033360.3 or NM_004985.4; *NRAS*: NM_002524.4 or LRG_92t1. Methods are ranked from least to more sensitive techniques as reported in literature: NGS 1–3%, commercial kit 0.1%, BEAMing 0.01%, ddPCR 0.001% [28]. For the LDT, a LOD of 0.1% was reported by the laboratory. Abbreviations: *BEAMing* Beads, emulsification, amplification, and magnetics, *ddPCR* Droplet digital polymerase chain reaction, *LDT* Laboratory-developed test, *NGS* Next-generation sequencing

### EQA sample preparation and validation

A panel of 10 artificial samples, 5 samples each for colorectal (Cases A-E) and lung (Cases F-J) cancer testing, were manufactured by and purchased from Horizon Discovery Ltd. (Cambridge, United Kingdom) according to a specification provided by the IQN Path working group. These included common, clinically relevant mutations in the *KRAS*, *NRAS* and *EGFR* genes with variant allelic frequencies of 1% or 5%, and also incorporated two wild-type samples (Table 1). Each sample comprised 3 mL human plasma containing 20 ng/mL ctDNA, fragmented to 150 base pairs in length.

Samples were created by reviving and expanding characterised cell lines of which gDNA pellets were created. DNA was extracted from the pellets, fragmented to 150 base pairs (+/− 10%), and diluted to the target concentration. The obtained cfDNA was spiked into normal human donor plasma, for which a copy detection analysis was performed on the background genes. The DNA was extracted once more and a final quality check was performed by estimating the fractional abundance.

Prior to their use in the pilot EQA scheme, each sample was characterised and validated by five reference laboratories, using a range of methodologies (Table 2) to verify sample performance in the pre-analytical and analytical processes, as well as to confirm that the expected genotype met the material specification provided by the IQN Path working group, and to ensure that the material reflected routine clinical samples in the hands of multiple laboratories. Extraction and analysis methods were selected based on the available methodologies that were validated for *EGFR* and/or *RAS* analysis in the reference laboratories, with the purpose of reflecting at least one method for every technique type, namely next-generation sequencing (NGS), droplet digital PCR (ddPCR), commercial kit, and beads, emulsification, amplification, and magnetics (BEAMing). Optionally, a second laboratory validated the samples using the same methodology if available. The analysed results from the validation trial were collectively reviewed by the IQN Path working group before the materials were released for use in the pilot EQA scheme.

**Table 2:** Overview of error rates per case for different methods for cfDNA extraction and variant analysis during validation

cfDNA extraction method	Cobas cfDNA sample preparation kit (Roche)	QIAamp Circulating Nucleic Acid Kit (Qiagen)					
Variant analysis method	Cobas® EGFR Mutation Test v2 (Roche)	Capture SureSelect (Agilent), MiSeq (Illumina)	QX200 Droplet Digital PCR System (Bio-rad)	Ampliseq 50 gene hotspot panel, Ion Proton (LifeTechnologies)	Therascreen® EGFR Plasma RGQ PCR Kit (Qiagen)	OncoBEAM® RAS CRC IVD KIT (Sysmex-Inostics)	
Reference laboratory code	1, 2	2	3°, 4	4	5	5	1–5
Sample	# errors/# genotypes analyzed (error rate in %)						
A	/	1/1 (100.0%)	0/2 (0.0%)	0/1 (0.0%)	/	0/1 (0.0%)	1/5 (20.0%)
B	/	1/1 (100.0%)	0/2 (0.0%)	1/1 (100.0%)	/	0/1 (0.0%)	2/5 (40.0%)
C	/	1/1 (100.0%)	0/1 (0.0%)	0/1 (0.0%)	/	0/1 (0.0%)	1/4 (25.0%)
D	/	1/1 (100.0%)	1/1 (100.0%)	1/1 (100.0%)	/	0/1 (0.0%)	3/4 (75.0%)
E	/	0/1 (0.0%)	0/2 (0.0%)	0/1 (0.0%)	/	0/1 (0.0%)	0/5 (0.0%)
F	0/2 (0.0%)	1/1 (100.0%)	0/2 (0.0%)	0/1 (0.0%)	0/1 (0.0%)	/	1/7 (14.3%)
G	0/2 (0.0%)	1/1 (100.0%)	0/2 (0.0%)	0/1 (0.0%)	0/1 (0.0%)	/	1/7 (14.3%)
H	0/2 (0.0%)	1/1 (100.0%)	0/2 (0.0%)	1/1 (100.0%)	0/1 (0.0%)	/	2/7 (28.6%)
I	1/2 (50.0%)	1/1 (100.0%)	1/2 (50.0%)	1/1 (100.0%)	0/1 (0.0%)	/	4/7 (57.1%)
J	0/2 (0.0%)	0/1 (0.0%)	0/2 (0.0%)	0/1 (0.0%)	0/1 (0.0%)	/	0/7 (0.0%)
A-J	1/10 (10.0%)	8/10 (80.0%)	2/18 (11.1%)	4/10 (40.0%)	0/5 (0.0%)	0/5 (0.0%)	15/58 (25.9%)

/, Sample not tested because gene not included in validated methodology. °Reference laboratory n°3 did not test *NRAS* status. Reference sequence at time of scoring: *EGFR*: NM_005228.4 or LRG_304t1; *KRAS*: NM_033360.3 or NM_004985.4; *NRAS*: NM_002524.4 or LRG_92t1

### Computational and statistical analysis

EQA participant and validation data from the pilot EQA scheme were analyzed using Microsoft Excel 2013 (Microsoft, Redmond, WA, United States of America). The overall error rate was calculated by dividing the total number of false-positive and false-negative results over the total number of genotypes reported by the participants. False-positive or false-negative results for which the treatment outcome would be affected were considered as critical errors when calculating the rate. Incorrect variants at the same codon were not classified as critical genotyping errors. False-negative results for which the sample genotype was not included in the methodology, or where it was below the stated limit of detection (LOD), were included in error rates because laboratories offering diagnostic mutational analysis on cfDNA should test for the selected clinically relevant variants. Technical failures were excluded from the total number of genotypes. Participants that did not subscribe and thus did not receive either the 5 *EGFR* or 5 *RAS* samples were also not scored for those samples. Statistical difference between reported variant allele frequencies (VAFs) were compared between techniques using a Mann Whitney U (MWU) test, for both the 1% and 5% variants, with a significance level of α = 0.05.

## Results

No technical failures were observed by the reference laboratories using two commonly used cfDNA extraction methods and six different mutation test methods (Table 2).

Fifteen (25.9%) false-positive or false-negative results were reported for a total of 58 analyzed genotypes (Table 2). On average, more false-negative results were reported for the *RAS* samples when compared to *EGFR*. The Capture SureSelect (Agilent) panel on the MiSeq (Illumina) sequencer was not able to detect any of the included *EGFR* or *RAS* variants in the plasma samples. In contrast, the Ion Ampliseq 50 gene hotspot panel on an Ion Proton (Life Technologies) was able to detect the single deletions in exon 19 of *EGFR* and *RAS* variants included at 5%. However, in the samples with *EGFR* p.(L858R) and p.(T790 M) (cases H, I), 6/14 tests were not able to detect at least one of the two mutations. No false-positive results were reported in the two wild-type samples or in any of the other cases as an additional variant.

The validation of these samples revealed that different ctDNA-based detection methods are able to correctly detect the genotype in 1% and 5% samples with a low false-positive rate. Our validation procedure also revealed that for less sensitive analytical methods, the 1% samples can be challenging. As the VAFs were still relatively high, we decided to perform the pilot EQA for *KRAS/NRAS* and *EGFR* using the five samples for the *EGFR* and *KRAS/NRAS* scheme. To additionally assess the quality of the samples, DNA yield was measured by each of the five reference laboratories using the QIAamp Circulating Nucleic Acid Kit (Qiagen), and resulted in an average of 0.66 μg/mL (min. 0.11 μg/mL, max. 4.63 μg/mL). Assessing the DNA yield is not part of the integrated workflow for cobas extraction and analysis.

**Fig. 1:**
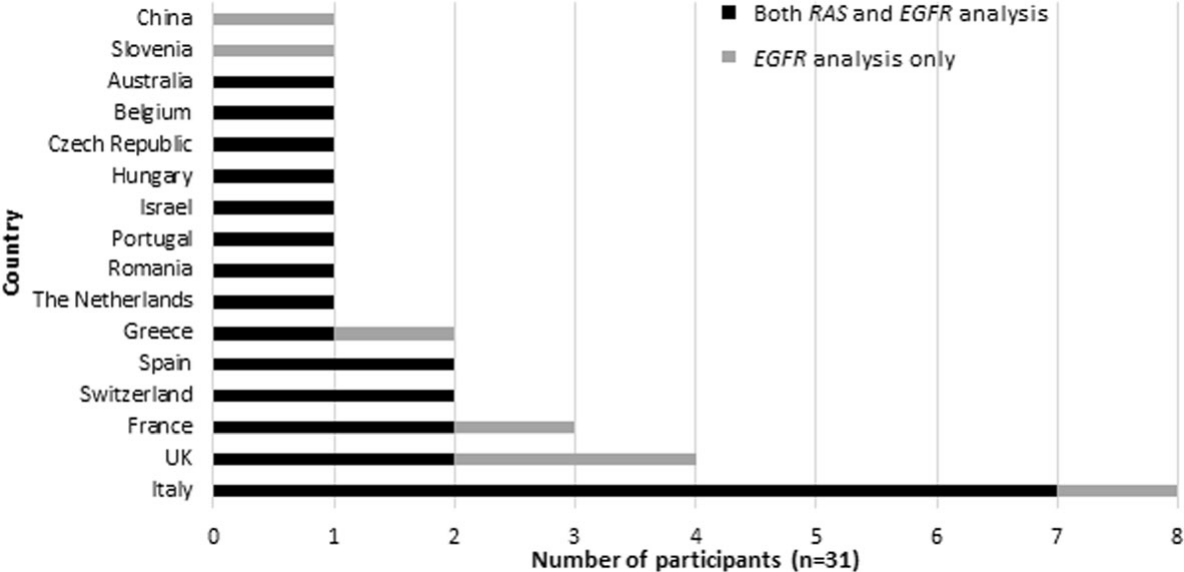
Overview of the participating countries to the pilot EQA scheme. United Kingdom: One laboratory received both *RAS* (*KRAS/NRAS) and EGFR* samples but did not submit results for *KRAS/NRAS* as they were in the process of validation. In total, 23 participants tested the samples for *KRAS/NRAS* analysis, and 31 participants for *EGFR* analysis

In total, 32 laboratories from 16 countries participated in the pilot EQA scheme (Fig. 1). Thirty-one (97%) laboratories submitted an electronic datasheet providing details on their cfDNA extraction, analysis methods, and a list of variants tested. One of the 31 laboratories did not submit written reports, therefore their genotyping results were only scored on the entries from the electronic table. In total, 23 participants tested the samples for *KRAS/NRAS* analysis, and 31 participants for *EGFR* analysis. Three of 23 participants receiving the *KRAS/NRAS* mutation samples did not perform any *NRAS* mutation testing but did perform *KRAS* analysis in these samples.

Of all 31 participants, six different cfDNA extraction methods were used (Table 3). The majority of the participants (55%) used the QIAamp Circulating Nucleic Acid Kit (Qiagen) for cfDNA extraction. Only one laboratory used an automated cfDNA extraction method (Promega Maxwell® RSC ccfDNA Plasma Kit). For *KRAS, NRAS* and *EGFR* mutation analysis, the most frequently used detection methodologies were NGS (39%) and droplet digital polymerase chain reaction (ddPCR) (23%) (Table 3, Additional file 2: Table S2). A combination of platforms and panels was applied, although the largest fraction of NGS users analyzed the plasma samples with the PGM Ion Torrent (Life Technologies).

**Table 3:** Overview of the cfDNA extraction and variant analysis methods methods used by the participants

	# participants to *KRAS* analysis (%) (*n* = 23)	# participants to *NRAS* analysis (%) (*n* = 20)	# participants to *EGFR* analysis (%) (n = 31)
cfDNA extraction method			
QIAamp Circulating Nucleic AcidKit (Qiagen)	14 (60.9)	13 (65.0)	17 (54.8)
Cobas cfDNA Sample Preparation Kit (Roche)	4 (17.4)	3 (15.0)	8 (25.8)
MagMAX Cell-Free DNA Isolation Kit (Thermo Fisher Scientific)	3 (13.0)	3 (15.0)	3 (9.7)
Maxwell® RSC ccfDNA Plasma Kit (Promega)	1 (4.3)	0 (0.0)	1 (3.2)
Nucleospin Plasma XS (Macherey-Nagel)	1 (4.3)	1 (5.0)	1 (3.2)
QIAamp DSP DNA Blood Mini Kit (Qiagen) version 2	0 (0.0)	0 (0.0)	1 (3.2)
Variant analysis method			
NGS	13 (56.5)	13 (65.0)	12 (38.7)
Commercial Kit	4 (17.4)	3 (15.0)	11 (35.5)
LDT	1 (4.3)	0 (0.0)	1 (3.2)
BEAMing	1 (4.3)	1 (5.0)	0 (0.0)
ddPCR	4 (17.4)	3 (15.0)	7 (22.6)

The *LDT* consisted of a 5’nuclease polymerase-chain reaction (Taqman) with peptide nucleic acid probe. For a detailed breakdown of the used methods see Additional file 2: Table S2. *Abbreviations*: *BEAMing* Beads, emulsification, amplification, and magnetics, *ddPCR* Droplet digital polymerase chain reaction, *LDT* Labroratory-developed test, *NGS* Next-generation sequencing

In total, 3 (1.1%) technical failures by 3 different participants, were observed from a total of 270 reported genotypes. One technical failure was classed as a partial failure as only the *NRAS* gene analysis failed to provde a reportable result. Hence the reported genotypes for this case were included for *KRAS* analysis, yielding a total of 268 analyzed samples. The reasons reported for technical failures included NGS read depth too low (Case A), problem with DNA extraction (Case G), or a defective cartridge for the partial failure (*NRAS* only, Case B). The overall scheme error rate was 54 (20.1%) on the total of 268 samples. Most errors were observed for *KRAS/NRAS* mutation testing (34/114, 29.8% samples, cases A-D), whereas error rates for *EGFR* analysis were lower (20/154 samples, 13.0%, cases F-I).

Combining the two samples containing a variant at a frequency of 5% and the two with a variant of 1%, yielded a total error rate of 15/45 (33.3%) and 19/46 (41.3%) compared to 4/61 (6.6%) and 15/62 (24.2%) for *RAS* and *EGFR* testing respectively. Sample I was withdrawn from the EQA scheme assessment but for information purposes, for *EGFR* analysis, the majority of the genotyping errors (13 of 20 errors) were observed for this sample I (Table 1) as only 18 out of 31 laboratories (58%) reported the presence of both the *EGFR* mutations at a frequency of 1%. Only one false-positive result (1/54 samples, 1.9%) was observed in the two wild-type samples (cases E and J). In the other four cases, 4/91 false-positive results were obtained for *RAS*, and 1/123 for *EGFR* analysis (Table 1).

Genotyping errors with no impact on therapeutic decisions were also observed but not included in the calculation of the error rate e.g. the detection of an incorrect *KRAS/NRAS* nucleotide variant resulting in a change within the same codon, or the incorrect annotation of the *EGFR* exon 19 deletion by NGS users (Table 1). Taking into account the number of laboratories using a specific methodology, the method specific error rate over all samples was the highest for NGS (23%) compared to ddPCR (15%) and commercial kits (15%) (data not shown).

**Fig. 2:**
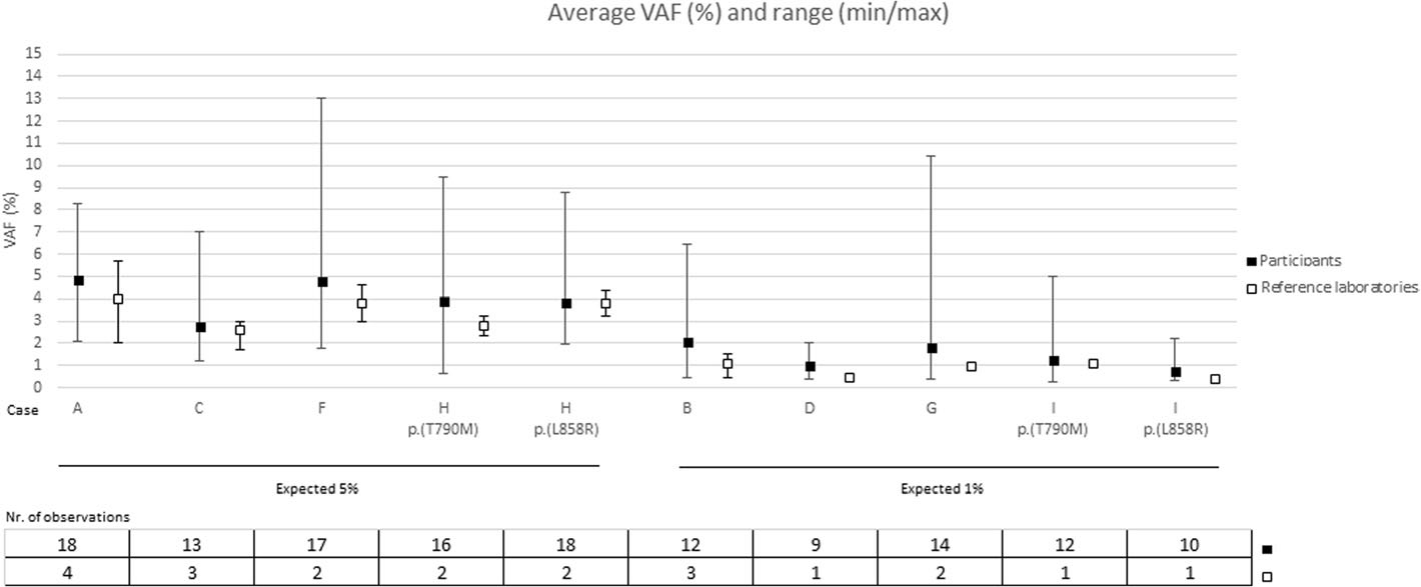
Average variant allele frequencies by the pilot scheme participants and reference laboratories. Case E and J were not included since they were wild-type. Only the variant allele frequencies of correctly identified variants were taken into account. Min: minimum variant allele frequency reported, max: maximum variant allele frequency reported

Participants were not asked specifically to report VAFs so only a small number of laboratories provided this information. The mean VAF was calculated for the cases containing a mutation, for which the mutation was correctly detected (Fig. 2). Average VAFs closely resembled the expected frequencies for 5% and 1%, but a very broad range was observed. The average VAF for the cases with variants at 5% was 4.0% (number of genotypes = 82, minimum VAF 0.6%, maximum VAF 13.0%). For variants at 1%, the estimated VAFs were 1.4% (number of genotypes = 57, minimum VAF 0.3%, maximum VAF 10.4%). (Fig. 2). Average VAFs were closer to the expected VAF for ddPCR when compared to NGS, but not significant for either the 1% cases (Mann-Whitney-U, *p* = 0.289, *n* = 11 ddPCR and *n* = 34 NGS) or the 5% cases (Mann-Whitney-U, *p* = 0.294, *n* = 17 ddPCR and *n* = 51 NGS).

The content of the reports varied between laboratories. The most important observation was that several laboratories over-interpreted the absence of a relevant mutation without providing information on quality control (QC) metrics. It is important to state if the input DNA and LOD were appropriate to reliably interpret the results as negative. Without this information, clinical interpretation may be incorrect. For example, a negative result could be interpretated as (i) the absence of a mutation indicating that the patient should receive anti-*EGFR* antibody therapy (in the case of CRC and *RAS* mutations) or that (ii) the patient would be unlikely to benefit from *EGFR*-tyrosine kinase Inhibitors (in the case of NSCLC and *EGFR* mutation). In addition, there was no standardisation in the reporting of the amount of cfDNA extracted, or the LOD. Only a small number of laboratories related the amount of input cfDNA to the assay sensitivity. Variation was also observed for several other elements, including the correct use of Human Genome Variation Society (HGVS) nomenclature [16, 17], reporting of reference sequences [18], and the specification of analysis limits of the methodology.

## Discussion

Plasma cfDNA analysis is emerging as a valuable tool to complement resected solid tumour or biopsy material in targeted treatment decisions. Many of the participating laboratories have been performing ctDNA analysis for some time. As there are no current EQAs for testing clinically relevant mutations in plasma, there is an urgent need for well-designed EQA schemes to provide education and benchmarking in order to permit implementation in an accurate, highly qualitative manner [13].

The acquisition and validation of artificial material for this pilot ctDNA molecular testing EQA was harmonized between several EQA schemes. The main goal was to harmonize the minimal requirements for the implementation of a ctDNA EQA scheme, in order to score the laboratories’ analytical performance and reporting, and eventually to serve as guidance for the organization of future large-scale EQA schemes. Secondly, harmonization between the four European EQA providers aimed to increase efficiency, and reduce the cost of delivery and speed of access to EQA.

This pilot EQA scheme demonstrated the feasibility of designing and delivering a technically challenging EQA. It also demonstrated sample stability during in-house distribution, preparation and transportation, which enabled the testing laboratory to produce a reportable result.

Technical failures were reported for only 3/270 (1.1%) of samples (Table 1) and there were none reported during validation (Table 2). However, a high rate of genotype errors was observed by the participants (20.1%). Prior to distribution, in the validation process we observed that the samples with 1% VAF and cases with the two relevant *EGFR* variants were challenging. This was reflected in FN rate (Table 1). Although during validation only six different detection methods were applied (including two different NGS assays), the results indicated that the analytical sensitivity of the methods is important and could be an explanation for the poorer performance of NGS.

In the pilot scheme the participants used a wide range of detection methods, and selected arbitrary cut-offs as a LOD for their assays (when indicated). Our analysis revealed that the highest error rates (false-negative rates) occurred for less sensitive techniques for ctDNA analysis, in concordance with the validation testing and the recent German pilot scheme [19]. Interestingly, when participants were separated into those using commercially available panels (*n* = 8 for both *EGFR* and *RAS* analysis) and those using in-house primers or panels (*n* = 5 for *RAS* and *n* = 4 for *EGFR* analysis), the commercial NGS methods showed excellent scores whereas the latter demonstrated a significantly higher error rate. These findings underline the need for robust validation of in-house NGS approaches for cfDNA testing.

For the samples which yielded a reportable result, more errors were observed for *RAS* analysis when compared to *EGFR* analysis. *EGFR* mutation testing in cfDNA is already widely implemented in clinical practice, whereas *RAS* plasma testing is still an experimental procedure in many centers, a fact which may account for the error rates. In addition, more participants are using commercial, targeted assays for *EGFR* detection compared to NGS for *RAS* analysis (Table 3). Non-NGS based methods are known to have a greater sensitivity and require less complicated bio-informatics. Despite the high error rate for case D, this sample was retained in the assessment as errors seemed to be related to a poorer performance of NGS, with more participants using this technology compared to *EGFR* analysis for case I. As recommended previously [19], we evaluated the estimated VAFs compared to the expected outcome, in order to assess scheme quality (Fig. 2). We found that average VAFs closely resembled the expected frequencies for 5 and 1%, especially for ddPCR when compared to NGS, although results were not significant as a broad range of VAFs were reported.

Many genotyping errors were observed for the two cases which included both an activating and a resistance *EGFR* variant: the majority of participants did not detect the p.(T790M) variant, especially at a VAF of 1%. Since the majority of *EGFR* mutations detected in the ctDNA of NSCLC patients are detected at < 5% allelic frequency, this would mean that a significant fraction of patients would not have received targeted treatment as result of these tests. For metastatic colorectal cancer, the likely consequence of a false negative result is that a patient inappropriately receives anti-*EGFR* treatment. The overall scheme error rate was higher than that observed in the German ctDNA EQA scheme [19]. However, we included variants at a VAF of 1% and 5% to resemble patient material as closely as possible, rather than at 5% and 10% as previously reported [19]. Furthermore, with the majority of laboratories using less sensitive techniques (Table 3), a fraction of the observed false-negative results occurred because the variant was included at a frequency below the LOD (Table 1). This observation highlights the issue of reporting mutations at low levels when the clinical significance is not known. Taking into account only the true false-negative results, the scheme error rates would be lower and therapy decision making would not always be compromised.

However, the error rates should be interpreted with some caution especially in assays used by a small number of participants, such as BEAMing and for some laboratory-developed tests (LDTs) (Table 3). To be able to draw firm conclusions on different cfDNA detection assays, an EQA with more than 500 participants is needed on a regular basis.

The high number of genotyping errors reported by this group of participants potentially indicates that the artificial material provided does not perform the same way as clinical samples. The difficulties in the implementation of this new methodology to clinical practice and the enormous variation in methods to process plasma, extract cfDNA and detect ctDNA, all compounded by a lack of guidelines, go some way to explain the observed variations. Additionally, some laboratories reported difficulties in extracting sufficient cfDNA material or specifically reported a reduced assay sensitivity due to the limitations of the supplied material.

Finding sufficient plasma samples from patients with known ctDNA mutations to use in EQA is challenging, mainly due to the amount of plasma required. For this reason, EQA providers are limited to using artificial EQA samples. In this pilot EQA scheme, cell-line derived DNA was spiked into normal plasma, which has the advantage that plasma quantities can be boosted. However, it also runs the risk that different background DNA levels could be present. The fact that cell-line DNA was used instead of plasmids has the advantage of allowing stoichiometric and unbiased dilutions, including QC of the dilution steps, as well as permitting fragmentation of the DNA to resemble the structure of ctDNA observed in patients. Alternatively, artificial plasma may be used [20]. However, plasmid DNA may not be an ideal control sample as it does not represent the true genomic complexity of human tumour samples [20].

Besides the analytical assessment, EQA also assesses the post-analytical phase. The pilot EQA scheme results stress the need for standardization of several elements. Although reporting has been shown to improve across subsequent EQA schemes for formalin-fixed paraffin-embedded tissue for different EQA providers [21, 22], plasma cfDNA testing as a new technology requires the inclusion of specific content in addition to some general elements, such as the use of standardized HGVS nomenclature [16, 17] and reference sequences [18]. However, best practice guidance for cfDNA reporting is currently not available.

More specifically, this pilot EQA highlighted the need to report wild-type results, and to provide a clinical interpretation when no mutation was detected. Because, even in samples where a mutation is present, there are several reasons why a wild-type result might have been obtained.

At certain stages of cancer progression, the amount of ctDNA may be too low to detect, as there is no shedding of tumour DNA. For CRC and NSCLC, a positive association has been described between the tumour volume and the presence of ctDNA [6, 23, 24]. In addition, whether the disease is localized rather than metastatic also significantly affects the ctDNA content in gastro-intestinal stromal tumours [25]. In only 70% of NSCLC cases, the *EGFR* mutation detected in the biopsy is also detected in plasma at the base-line [26] and at progression while on therapy [3]. Therefore, in the case of negative results with sufficient cfDNA input, it is important to obtain a tissue biopsy and when this is not possible, plasma testing should be repeated on a new sample. We also recommend not to use the terms ‘positive/negative’ to describe the mutation status in reports, as this can be misinterpreted: rather, ‘mutation detected/mutation not detected’ terminology should be employed. Secondly, a false-negative result can arise if the sensitivity and LOD of the assay is too low, and to date assay sensitivities vary between < 0.1 - < 1% [4]. Therefore, data sensitivity of mutation detection and LOD should be recorded in the report. In the pilot scheme there was a high diversity among laboratories regarding the reporting of sensitivities, which were expressed in either as copies/mL or as allelic frequency (percentage). For both options it is recommended that the amount of cfDNA extracted for a sample is included and that this should be related to the assay sensitivity because if the input of the total amount of cfDNA is too low, the test will also be negative. Thirdly, if the assay does not cover all the relevant variants and regions, a mutation might be missed. Therefore, a detailed inclusion of the list of variants, codons or exons tested should be present.

It is important to report the QC metrics of the test performance. Several laboratories reported an incorrect sequence of the deletions in *EGFR* exon 19. While this error will not compromise patients’ treatment, it highlights the need for improvements of bioinformatics workflow. A false-negative result could also arise due to haemolysis during collection and processing of blood plasma, diluting the mutant DNA to non-detectable levels [3, 27]. Therefore it is clear that ctDNA testing requires additional guidelines for preanalytical processing.

The utility of circulating biomarkers in the molecular analysis of solid tumours is an exciting new mutation detection tool with many potential applications [28]. However, the highly sensitive testing technology and the handling of appropriate samples is challenging. Standardization is essential to ensure that patients receive the correct results, and so that appropriate treatment is delivered. The provision of EQA is also essential to reassure testing laboratories of the standard of their cfDNA testing service.

## Conclusions

As with all EQA schemes, laboratories are encouraged to review their EQA results to ensure no errors have occurred. Errors can impact on the clinical testing service by following up on sub-optimal performance. Based on the findings of this pilot EQA scheme, the need for EQA schemes for all laboratories providing a cfDNA mutation testing service for lung and colorectal cancer has been identified. With this in mind, a second EQA round will be organized in 2018, which will be open to all laboratories from all countries.

## Additional files

Additional file 1: Example of individual feedback report. (PDF 136 kb)

Additional file 2: **Table S2.** Description: Detailed overview of the mutation detection techniques used by the EQA participants. (XLSX 12 kb)

## Abbreviations

AIOM: Associazione Italiana di Oncologia Medica
BEAMing: Beads, emulsification, amplification, and magnetics
cfDNA: Circulating cell-free DNA
CRC: Colorectal cancer
ctDNA: Circulating tumour DNA
ddPCR: Droplet digital polymerase chain reaction
*EGFR*: Epidermal growth factor receptor
EMQN: European Molecular Quality Network
EQA: External quality assessment
ESP: European Society of Pathology
HGVS: Human Genome Variation Society
IQN Path: International Quality Network for Pathology
*KRAS*: Kirsten rat sarcoma viral oncogene homolog
LDT: Laboratory-developed test
LOD: Limit of detection
NGS: Next-generation sequencing
*NRAS*: Neuroblastoma rat sarcoma viral oncogene homolog
NSCLC: Non-small-cell lung cancer
QC: Quality control
UKNEQAS: United Kingdom National External Quality Assessment Service
VAF: Variant allele frequency
