# Supplementary material for: International pilot external quality assessment scheme for analysis and reporting of circulating tumour DNA
Source: BMC Cancer. 2018 Aug 9;18:804. doi: 10.1186/s12885-018-4694-x (PMC6085634; doi:10.1186/s12885-018-4694-x)
Supplement: Supplementary file 1 — Example of individual feedback report. (PDF 136 kb) [file 12885_2018_4694_MOESM1_ESM.pdf]

INDIVIDUAL LABORATORY REPORT (ILR) - Lab XXX

SCHEME: IQNPath Liquid Biopsy EQA (pilot) **SEASON:** 2017

IQNPath Liquid Biopsy EQA (pilot) Case 1 RAS

Assessment Category	Score[1]	Comments (& deductions[2])
**Genotyping**	2.00	Correct result reported (method enables mutation characterisation AND correct HGVS nomenclature used) (0)<br>Comment: Reporting variants on genes that were not requested for testing
**Interpretation**	--	All essential interpretative elements provided<br>Mutations tested were not specified
**Patient Identifiers and Clerical Accuracy**	2.00	Comment(s) (0)<br><br>No pagination i.e. 1 of 2, 2 of 2 etc. A one page report is preferred.

IQNPath Liquid Biopsy EQA (pilot) Case 2 RAS

Assessment Category	Score[1]	Comments (& deductions[2])
**Genotyping**	2.00	Correct result reported (method enables mutation characterisation AND correct HGVS nomenclature used) (0)
**Patient Identifiers and Clerical Accuracy**	2.00	No deductions (0)

IQNPath Liquid Biopsy EQA (pilot) Case 3 RAS

Assessment Category	Score[1]	Comments (& deductions[2])
**Genotyping**	2.00	Correct result reported (method enables mutation characterisation AND correct HGVS nomenclature used) (0)
**Patient Identifiers and Clerical Accuracy**	2.00	No deductions (0)

IQNPath Liquid Biopsy EQA (pilot) Case 4 RAS

Assessment Category	Score[1]	Comments (& deductions[2])
**Genotyping**	2.00	Correct result reported (method enables mutation characterisation AND correct HGVS nomenclature used) (0)
**Patient Identifiers and Clerical Accuracy**	2.00	No deduction (0)

IQNPath Liquid Biopsy EQA (pilot) Case 5 RAS

Assessment Category	Score[1]	Comments (& deductions[2])
**Genotyping**	2.00	Correct result reported (method enables mutation characterisation AND correct HGVS nomenclature used) (0)
**Interpretation**	--	No interpretation provided<br>Mutations tested were not specified<br>It is recommended to state that the analysis of a plasma sample is not 100% sensitive and therefore the presence of a mutation may have been missed.

[1] Maximum score is 2.00
[2] Deductions from the maximum score
[3] Green >= Scheme mean, Orange < Scheme mean, Red Poor performance.
NRS no results submitted. WDS withdrew from scheme

Published: 22 June 2017

**Patient Identifiers and Clerical Accuracy**	2.00	No deduction (0)

**IQNPath Liquid Biopsy EQA (pilot) Case 1 EGFR**		
**Assessment Category**	**Score[1]**	**Comments (& deductions[2])**
**Genotyping**	1.00	Mutation described incorrectly e.g. incorrect deletion reported at nucleotide or amino acid level (non-critical genotyping error) (1)<br>The variant should be EGFR c.2235_2249del p.(Glu746_Ala750del).
**Interpretation**	--	No interpretation provided
**Patient Identifiers and Clerical Accuracy**	2.00	No deduction (0)

**IQNPath Liquid Biopsy EQA (pilot) Case 2 EGFR**		
**Assessment Category**	**Score[1]**	**Comments (& deductions[2])**
**Genotyping**	1.00	Mutation described incorrectly e.g. incorrect deletion reported at nucleotide or amino acid level (non-critical genotyping error) (1)<br>The variant should be EGFR c.2235_2249del p.(Glu746_Ala750del).
**Patient Identifiers and Clerical Accuracy**	2.00	No deduction (0)

**IQNPath Liquid Biopsy EQA (pilot) Case 3 EGFR**		
**Assessment Category**	**Score[1]**	**Comments (& deductions[2])**
**Genotyping**	2.00	Correct result reported (method enables mutation characterisation AND correct HGVS nomenclature used) (0)
**Patient Identifiers and Clerical Accuracy**	2.00	No deduction (0)

**IQNPath Liquid Biopsy EQA (pilot) Case 4 EGFR**		
**Assessment Category**	**Score[1]**	**Comments (& deductions[2])**
**Genotyping**	--	Correct result reported (method enables mutation characterisation AND correct HGVS nomenclature used)<br>Not marked<br>This sample contained two mutations in EGFR at an allele frequency of approximately 1% (confirmed by our validation studies). Only 18 laboratories (56%) reported the presence of both these mutations in this sample and therefore it was decided to exclude this case from the marking.
**Interpretation**	--	Comment(s)<br>The interpretation is inadequate rather than incorrect.It should be stated that the combination of the p.L858R and p.T790M mutations will lead to a shortened duration of response to EGFR TKIs. However, the patient may benefit to future treatment with 3rd generation TKIs.
**Patient Identifiers and Clerical Accuracy**	--	Not marked

**IQNPath Liquid Biopsy EQA (pilot) Case 5 EGFR**		
**Assessment Category**	**Score[1]**	**Comments (& deductions[2])**
**Genotyping**	2.00	Correct result reported (method enables mutation characterisation AND correct HGVS nomenclature used) (0)
**Interpretation**	--	No interpretation provided<br>Comment(s)<br>It should be stated that a negative result could be due to lack of sensitivity, and therefore that a biopsy should be considered.
**Patient Identifiers and Clerical Accuracy**	2.00	No deduction (0)

[1] Maximum score is 2.00
[2] Deductions from the maximum score
[3] Green >= Scheme mean, Orange < Scheme mean, Red Poor performance.
NRS no results submitted. WDS withdrew from scheme

Published: 22 June 2017

General Comments	General comment: No pagination i.e. 1 of 2, 2 of 2 etc. A one page report is preferred.<br><br>Thank you for participating in this pilot EQA scheme. We look forward to welcoming you to our meeting in Florence and hearing your feedback.

SUMMARY OF YOUR PERFORMANCE IN THIS SCHEME

Assessment Category	Performance[3] (mean score)
Genotyping	1.78
Interpretation	Not marked
Patient Identifiers and Clerical Accuracy	2.00
**Scheme result** (SATISFACTORY or POOR)	SATISFACTORY

NOTE: The results above are subject to change depending on the results of the appeals process.

The final results will be printed on the certificate of participation.

Report authorized by the head of the EQA provider on 22 June 2017.

[1] Maximum score is 2.00
[2] Deductions from the maximum score
[3] Green >= Scheme mean, Orange < Scheme mean, Red Poor performance.
NRS no results submitted. WDS withdrew from scheme

Published: 22 June 2017
